# Functional Evaluation of a Bioartificial Liver Support System Using Immobilized Hepatocyte Spheroids in a Porcine Model of Acute Liver Failure

**DOI:** 10.1038/s41598-017-03424-2

**Published:** 2017-06-19

**Authors:** Ji-Hyun Lee, Doo-Hoon Lee, Sanghoon Lee, Choon Hyuck David Kwon, Jae-Nam Ryu, Jeong-Kwon Noh, In Keun Jang, Hey-Jung Park, Hee-Hoon Yoon, Jung-Keug Park, Young-Jin Kim, Sung-Koo Kim, Suk-Koo Lee

**Affiliations:** 1 Stem Cell and Regenerative Medicine Center, Research Institute for Future Medicine, Samsung Medical Center, Seoul, Republic of Korea; 2 Department of Health Sciences and Technology, SAIHST, Sungkyunkwan University, Seoul, Republic of Korea; 3 Biomedical Research Center, Lifeliver, Co. Ltd., Yongin, Republic of Korea; 4 Department of Surgery, Samsung Medical Center, Sungkyunkwan University School of Medicine, Seoul, Republic of Korea; 5 Department of Chemical and Biochemical Engineering, Dongguk University, Seoul, Republic of Korea; 6 Seoul Clinical Laboratories, Yongin, Republic of Korea; 7 Department of Biotechnology and Bioengineering, Pukyong National University, Busan, Republic of Korea

## Abstract

Bioartificial livers (BAL) may offer acute liver failure (ALF) patients an opportunity for cure without liver transplantation. We evaluated the efficacy of a spheroid-based BAL system, containing aggregates of porcine hepatocytes, in a porcine model of ALF. ALF pigs were divided into three groups. The control group consisted of treatment naïve pigs (n = 5), blank group consisted of pigs that were attached to the BAL system not containing hepatocytes for 12 hours (n = 5) and BAL group consisted of pigs that were attached to the BAL containing hepatocytes for 12 hours (n = 5). Increase in serum ammonia levels were significantly greater in the blank group (P < 0.01) and control group (P < 0.01), compared to the BAL group during the treatment period. Increase in ICP was significantly greater in the control group compared to the BAL group (P = 0.01). Survival was significantly prolonged in the BAL group compared to the blank group (P = 0.03). A BAL system with a bioreactor containing hepatocyte spheroids showed effective clearance of serum ammonia, preservation of renal function and delayed ICP increase in a porcine model of ALF.

## Introduction

Although there have been significant advances in medical therapy, many patients with acute liver failure (ALF) die due to the sudden deterioration in liver function and complications associated with this condition. The introduction of liver transplantation (LT) as a therapeutic option for patients with ALF has increased survival rates, but limited organ availability enables only a small proportion of those affected by ALF to undergo timely transplantation^1, 2^.

Bioartificial liver (BAL) support offers a potential means of improving survival of ALF patients by providing partial liver function until a suitable donor liver is found or the native liver undergoes regeneration. Previous studies have suggested that ALF patients treated using BAL maintain a more stable medical condition, which may positively influence outcome after LT^3^.

Majority of BAL systems consist of a hollow fiber cartridge packed with single hepatocytes. However, in previous *in vitro* studies, hepatocyte spheroids were found to have greater liver-specific functions than dispersed single hepatocytes, especially, in terms of detoxification of plasma and serum^4–6^. We developed a BAL system using porcine hepatocyte spheroids immobilized on calcium-alginate beads, which we believe is a more effective BAL system compared to hollow fiber-based BAL systems. The aim of this study was to evaluate the efficacy of an aggregated porcine hepatocyte BAL system in a porcine model of ALF.

## Results

**Figure 1:**
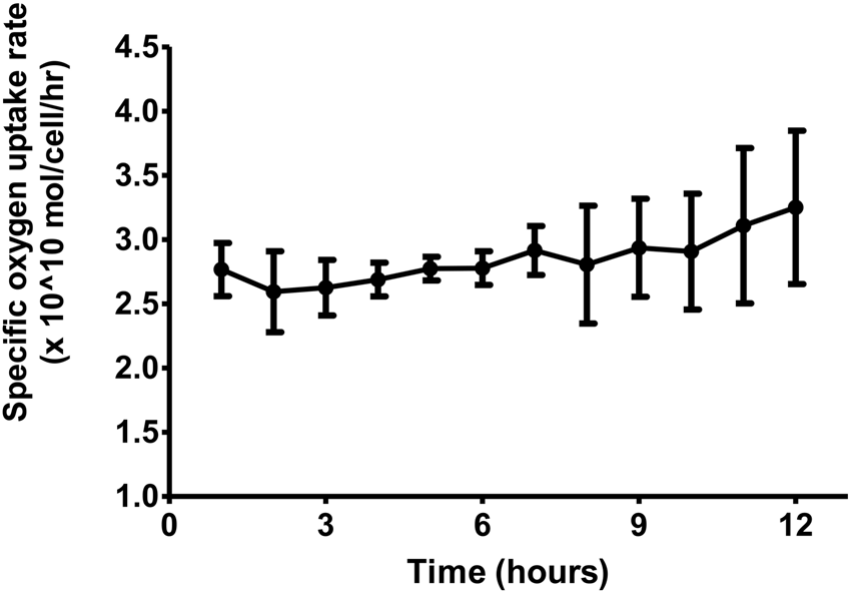
Specific oxygen uptake rate of the alginate-encapsulated hepatocytes in BAL group (n = 5). Gas was supplied right before the reactor, comprised of 95% oxygen and 5% carbon dioxide. Data of dissolved oxygen was measured in real-time before and after the reactor. The flow rate of plasma in the reactor was 250 ml/min. Specific oxygen uptake rate decreased slightly during the first hour of circulation then increased after 7 hours onward, signifying that the hepatocytes remained vigorous activity during the 12-hour treatment period.

Mean yield of hepatocytes from a single porcine liver was 2.48~4.26 × 10^10^ cells and average viability was 88% by trypan blue exclusion staining. Hepatocyte spheroids formed after culturing for 10 hours were used to produce calcium-alginate beads of 1000 ± 200 µm. These were then used to fill the bioreactor, which resulted in a bioreactor loading of 2.0 × 10^10^ viable immobilized hepatocytes (n = 5). Functional performance of the BAL system as shown by the specific oxygen uptake rate of hepatocyte spheroids remained unchanged throughout the 12-hour procedure (Fig. 1). Surgical induction of ALF was done in 15 pigs. Five pigs received standard intensive care (control group), 5 received BAL treatment (BAL group) and 5 received cell-free BAL treatment (blank group).

### Systemic response to ALF and survival

**Figure 2:**
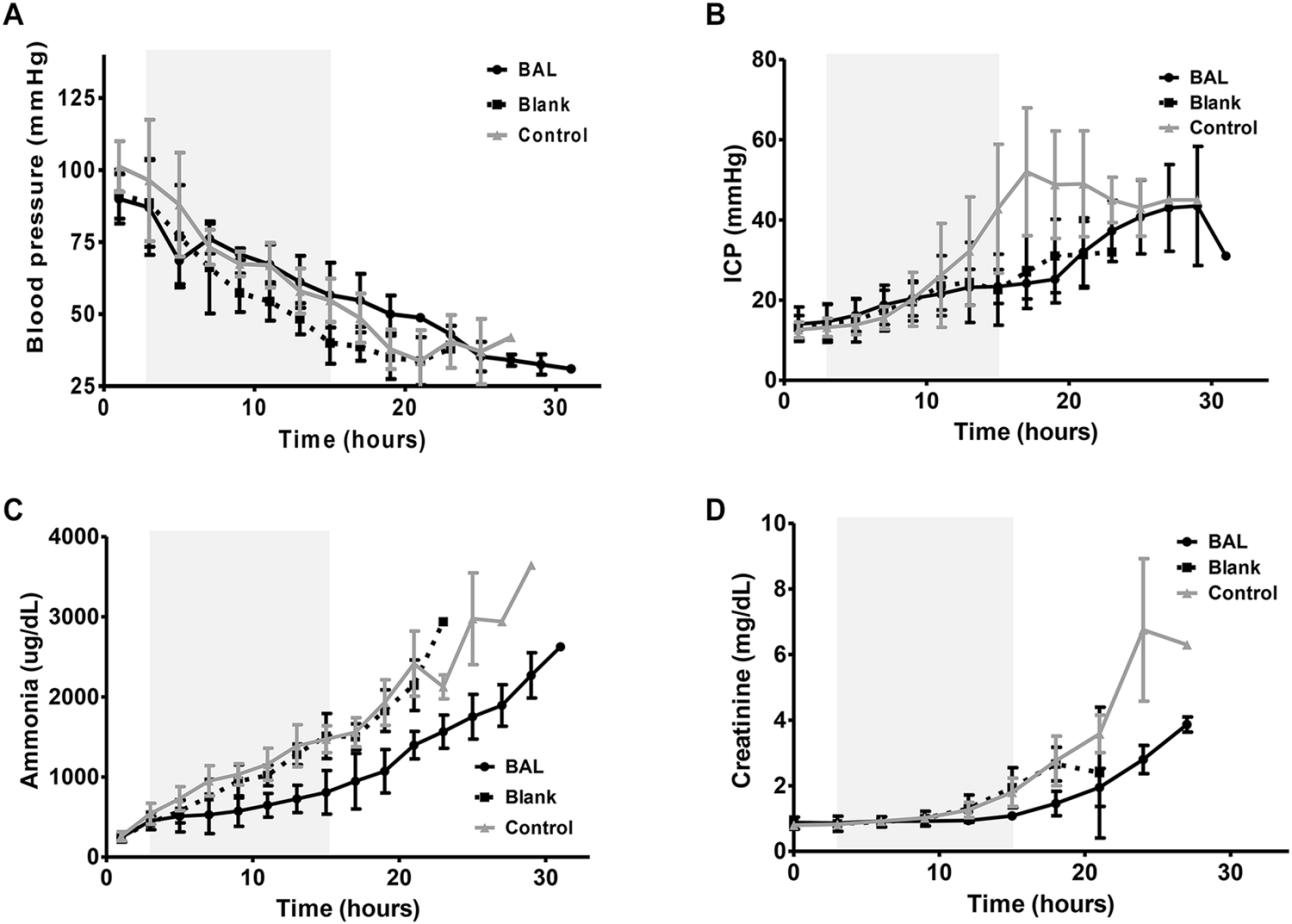
(**A**) Systolic blood pressure, (**B**) intracranial pressure, (**C**) serum ammonia concentration and (**D**) serum creatinine after induction of acute liver failure in pigs in the BAL group (black line), blank group (dotted line) and control group (grey line). Extracorporeal circulation periods are shaded grey.

Systemic blood pressure (BP) gradually decreased throughout the treatment period in all three groups, while the decrease in BP was significantly slower in the BAL group compared to the blank group (P = 0.02) and control group (P = 0.04, Fig. 2A). ICP gradually increased after induction of ALF in all animals, while the increase in ICP was significantly greater in the control group compared to the BAL group (P = 0.01, Fig. 2B). Trends in ICP were not different between the BAL group and blank group during treatment (Fig. 2B). Urine output gradually decreased during the treatment period in all three groups. The rate of progression of oliguria was more rapid in the blank group compared to the BAL group (P = 0.02), but not different between the control group and the BAL group (P = 0.91).

**Figure 3:**
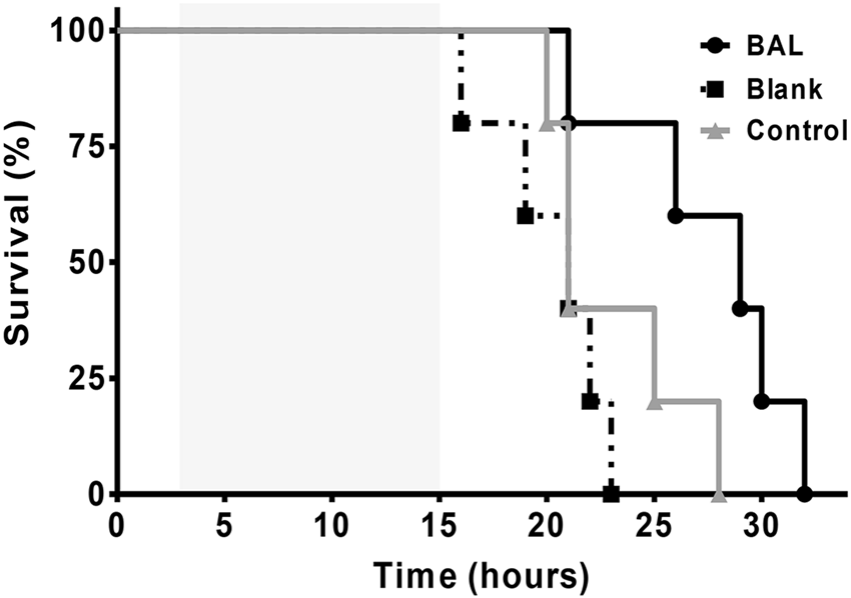
Kaplan-Meier survival curves showing significantly prolonged survival in the BAL group (black line) compared to the blank group (dotted line, P = 0.03). There was no significant difference in survival between the control group (grey line) and BAL group (P = 0.44). The 12-hour treatment period is shaded grey.

Median survival time of pigs in the BAL group, blank group, control group were 28.5 hours (95% C.I. 25~31 hours), 21 hours (95% C.I. 16~23 hours) and 21 hours (95% C.I. 20~28 hours), respectively. Kaplan-Meier survival analysis showed that survival was significantly prolonged in the BAL group compared to the blank group (P = 0.03, Fig. 3). There was no significant difference in survival between the control group and BAL group (P = 0.44).

### Biochemical parameters

**Figure 4:**
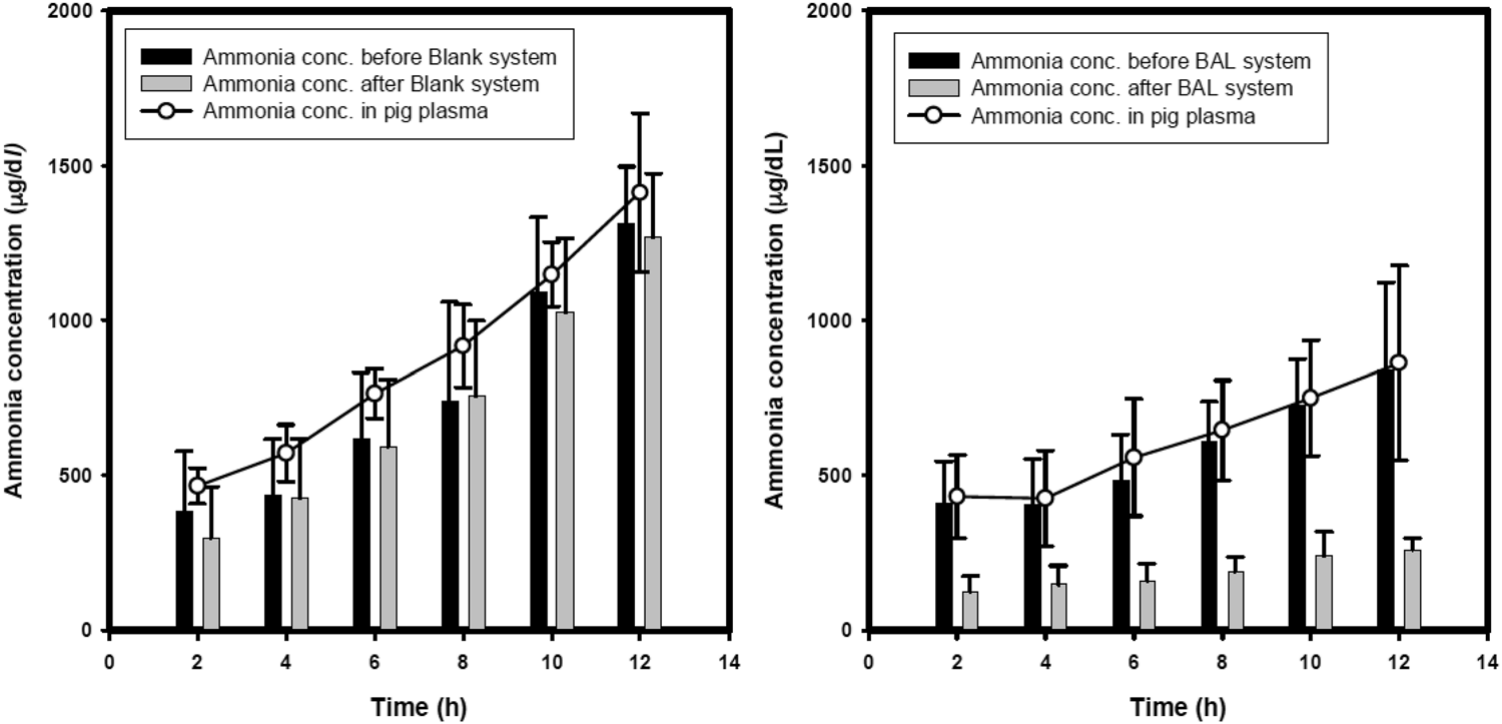
Ammonia levels recorded in the extracorporeal circulation in the (**A**) blank group and (**B**) BAL group during 12-hour treatment. Significant decrease in ammonia in the plasma exiting the extracorporeal circulation system (grey bars) compared to the plasma entering the extracorporeal circulation system (black bars) is observed in the BAL group (**B**).

Increase in serum ammonia levels were significantly greater in the blank group (P < 0.01) and control group (P < 0.01), compared to the BAL group during the treatment period after induction of ALF (Fig. 2C). Concentration of ammonia measured in the plasma entering and exiting the extracorporeal circulation system are shown in Fig. 4. Significantly lower concentrations of ammonia are detected in the plasma exiting the extracorporeal circulation system throughout the 12-hour treatment period in the BAL group compared to the blank group.

Serum creatinine levels remained significantly lower in the BAL group compared to the other two groups (P = 0.01 vs. blank group, P = 0.03 vs. control group) despite a decrease in intravascular volume due to the extracorporeal circulation system (Fig. 2D). Other biochemical parameters including AST, ALT, LDH, glucose, albumin and pH were not significantly different between the three groups.

No major technical problems were encountered during plasmapheresis or BAL application. No evidence of hemolysis was observed in the animals in the treatment group.

## Discussion

The principal finding of this study is that during 12 hours of treatment with a BAL system using immobilized hepatocyte spheroids in surgically-induced porcine model of ALF, survival was significantly prolonged compared to pigs receiving liver support treatment without hepatocytes. Superior serum ammonia clearance and suppression of ICP increment were also seen in pigs receiving BAL treatment. These results are evidence supporting the hypothesis that our BAL system would be able to effectively remove plasma toxins and facilitate control of increase ICP, leading to prolonged survival in ALF.

The BAL system used in the present study has unique features. First, its functional units are composed of spheroid hepatocytes rather than single hepatocytes, and it has been previously reported that spheroids have better survival and detoxification abilities^5, 6^. In an *in vitro* test previously performed, the viability of spheroids remained unchanged after 60 hours of circulation (data not shown). Our oxygen consumption results show that the functional performance of the BAL system remained unchanged throughout the 12-hour treatment period (Fig. 1). The second feature is that encapsulation of spheroids in calcium alginate beads has an advantage over hollow fiber cartilage BAL systems because the beads can be packed in tightly within a bioreactor, which increases the number of functioning hepatocytes per given volume. The cell concentration of our bioreactor is 3.3 × 10^7^ cells/ml, whereas the spheroid-reservoir bioreactor in the study by Glorioso *et al*. was 1~2 × 10^7^ cells/ml^7^. In a fluidized system, settling space of the upper part of reactor is required for cell or bead retention, which increases the reactor size. The space should eventually be filled with the patient’s plasma, leading to an added extracorporeal circulatory burden. Furthermore, the encapsulation in calcium alginate beads prevents hepatocytes from coming in direct contact with the animal’s circulating antibodies, which minimizes cell damage but allows uninhibited dissolution of toxins and harmful cytokines.

Increase in ICP was suppressed during treatment in the BAL group compared to the control group. However, this alleviation effect of ICP increase was also seen in the blank group. One possible explanation for this is that effective intravascular volume was more reduced in the blank and BAL groups than in the control group due to the extracorporeal circulation. Brain edema in ALF is the result of a combination of osmotic, metabolic, and hemodynamic changes. Reduced cerebral blood flow from a decrease in intravascular volume may contribute to decreased ICP^8–10^. We used 10% pentastarch to treat loss of blood volume from the extracorporeal circulation in both the blank and BAL groups. Hypertonic saline hydroxyethyl starch 7.55% has been reported to reduce ICP effectively^11^. However, a steeper increment in ICP was observed after discontinuation of treatment in the blank group compared to the BAL group, which indicates that BAL may have conferred an additional benefit.

Adequate liver function plays a key role in maintaining kidney function. Renal dysfunction is one of the clinical signs that appear during ALF. We observed a slower rise in serum creatinine and better urine output in the BAL group compared to the blank group and control group. However, decrease in urine output was not different between the BAL group and the control group. We believe this loss of additional benefit in preservation of kidney function is associated with the intravascular volume loss occurring during BAL treatment due to the extracorporeal circulation. Conversely, despite this reduced intravascular volume in the BAL group versus the control group, serum creatinine levels increased more slowly in the BAL group. This shows that the BAL system contributed to maintaining stable liver function and in turn, protected renal function enough to neutralize the negative effect of loss in intravascular volume.

In conclusion, a BAL system with a bioreactor packed with gel beads containing hepatocyte spheroids perfused by gravity driven flow of media was applied to a porcine model of ALF. This BAL system showed effective clearance of serum ammonia, preservation of renal function and delayed increase in ICP.

## Methods

### Animals

Small male pigs grown in clean barrier facilities (3~4 weeks old, mixed strain of Landrace, Yorkshire and Duroc weighing 4~10 kg) were used for hepatocyte isolation and large male pigs (3 months old, conventional farm pigs weighing 45~55 kg) were used as models of hepatic failure. This study was reviewed and approved by the Institutional Animal Care and Use Committee (IACUC) of Samsung Biomedical Research Institute (SBRI) and all experiments described in this section were performed in accordance with the guidelines and regulations of the committee. SBRI is an Association for Assessment of Accreditation of Laboratory Animal Care International (AAALAC International) accredited facility and abide by the Institute of Laboratory Animal Resources (ILAR) guidelines.

### Porcine hepatocyte isolation

Hepatocytes were harvested from 3~4 weeks old male pigs weighing 4 to 10 kg. Hepatocyte preparation was conducted using a modification of the two-step collagenase perfusion technique originally described by Seglen for rat hepatocytes^12^. In brief, with the animal under general anesthesia, the liver was perfused via the portal vein with 8 L of a warm oxygenized perfusion buffer (NaCl 8 g/l, KCl 0.4 g/l, NaH_2_PO_4_·2H_2_O 0.06 g/l, Na_2_HPO_4_·12H_2_O 0.06 g/l, glucose 2.7 g/l, NaHCO_3_ 1.05 g/l, HEPES 2.38 g/l, EDTA 0.19 g/l) at a flow rate of 80 mL/min/kg of body weight. 1000 ml of a solution containing calcium was then introduced through the portal vein to ensure optimal collagenase function. Finally, liver digestion was accomplished by recirculating 1500 ml of a perfusion buffer supplemented with collagenase (0.3 g/l) and calcium chloride (0.735 g/l) at a flow rate of 70 mL/min/kg of body weight at 37 °C for about 8 min, to break down the extracellular matrix. When the liver softened and the capsule began to rupture spontaneously, the collagenase solution was immediately removed, and the digestion process was stopped with 1000 mL of cold (4 °C) Williams E medium (Sigma). The liver was then cooled in a sterile container on ice, minced, and gradually filtered through a mesh (pore size 500 to 150 μm). Cells were then pelleted and cell suspensions were washed and centrifuged three times at 50 × g for 4 minutes at 4 °C. Trypan blue exclusion staining showed that the hepatocytes so obtained were more than 85% viable.

### Spheroid culture and calcium alginate immobilization

Hormonally defined medium (HDM) was used to culture spheroids. It was composed of William’s E medium supplemented with 20% albumin (5 mL/l), epidermal growth factor (EGF, 20 μg/l), insulin (10 mg/l), CuSO_4_·5H_2_O (24.97 μg/l), ZnSO_4_·7H_2_O (14.38 pg/l), H_2_SeO_3_ (3 μg/l), NaHCO_3_ (1.05 g/l), HEPES (1.19 g/l), penicillin (58.8 mg/l) and streptomycin (0.1 g/l).

Briefly, isolated hepatocytes were inoculated at a cell density of 1.5 × 10^6^ cells/ml in a spinner flask containing 2000 ml of culture medium and stirred with a magnetic stirrer at 20 rpm. Gas-phase of the spinner flask was purged with 95% O_2_ and 5% CO_2_ gas to support high oxygen demand of hepatocytes. Twenty billion hepatocytes were cultured for 10 hours to form spheroids with 70 μm mean diameter. Cell viabilities were reassessed by trypan blue dye exclusion and MTT conversion before encapsulation. Viable hepatocyte spheroids were then mixed with 1.5% alginate solution, which had been previously heat-treated to ensure dissolution and sterilization. This mixture was then placed in a high content/speed immobilization apparatus and dropped into 100 mM calcium solution.

### Bioreactor, gravity fed perfusion system and extracorporeal BAL circulation system

**Figure 5:**
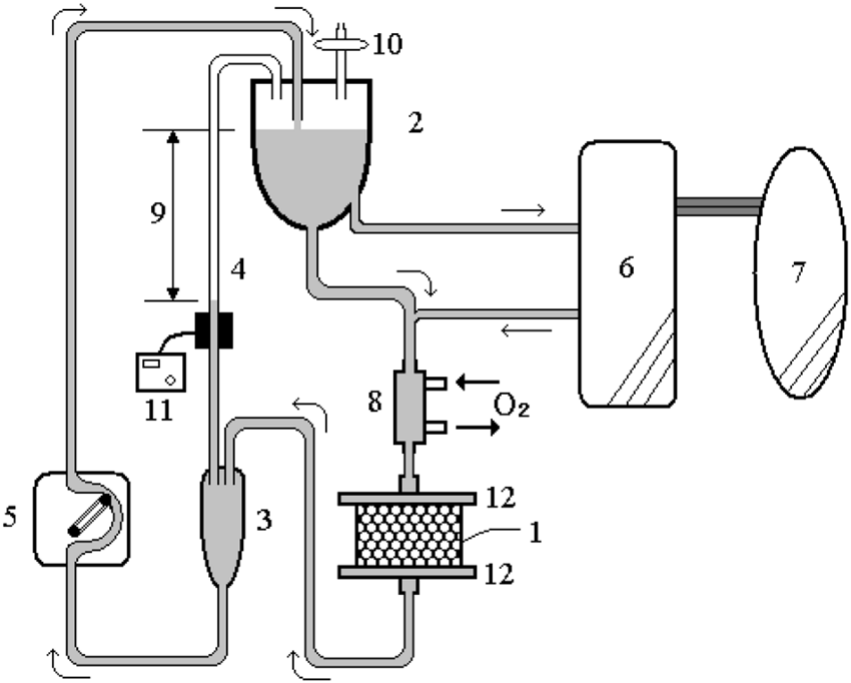
Schematic illustration of the gravity fed perfusion system used in this study. Separated plasma flow into the system and later return to blood. Treatment was started 3 hours after total liver ischemia. Vascular access was achieved using a double lumen catheter inserted into the internal jugular vein. The plasma is passed through the bioartificial liver (BAL) system and recirculate at 300 ml/min through the reservoir, an oxygenator/heater and the bioreactor. After recirculation through the BAL system, plasma and red blood cells are reconstituted in the plasmapheresis system and returned by way of a dual-lumen venous catheter. (1: bioreactor, 2: reservoir, 3: chamber, 4: line, 5: pump, 6: plasma separator, 7: anhepatic pig, 8: oxygenator & heater, 9: gravity force, 10: air filter, 11: air detector, 12: mesh).

The packed-bed bioreactor contained Ca-alginate-immobilized hepatocyte spheroids beads. Its housing was constructed from polycarbonate and packing volume was approximately 600 ml. A plasma distributor was placed on top of the bioreactor to achieve even flow. The perfusion system was composed of blood circulation tubing, a pump, a reservoir, an oxygenator, a heat exchanger and mesh (Fig. 5). The perfusion pressure against the packed-bed bioreactor was limited to 30 mmHg, which was equivalent to the maximum pressure drop generated by 40 cm media level difference between the reservoir and the outlet chamber. Perfusion flow was set using a peristaltic pump within the flow rate constraints determined by outlet chamber location.

In the present study, a clinical prototype of the BAL system was used. Before use, each blood circuit and the bioreactor were washed with 2 L of heparinized saline, which was used as an anticoagulant to prevent thrombosis within the system. Activated clotting time (ACT) was measured hourly during operation and maintained between 160–180 seconds. Access and return lines were connected to the pig’s internal jugular vein using a dual-lumen catheter. Blood was continuously drawn (100 ml/min) and separated into cellular elements and plasma using an automated plasmapheresis system (Spectra, COBE BCT, Lakewood, CO). Plasma then entered the BAL system at 40 ml/min, and recirculated at 300 ml/min through the reservoir, the oxygenator/heater, and the bioreactor. After recirculation through the BAL system, plasma and red blood cells were reconstituted in the plasmapheresis system and returned to the animal using a double-lumen venous catheter. BAL treatment started 3 h after completing the surgical procedure, described below. Only one bioreactor of immobilized porcine hepatocytes spheroids was used during the entire procedure. Filters were integrated into plasma lines to and from the bioreactor to remove blood cells.

### Experimental acute liver failure porcine model

The study was performed on healthy pigs weighing 45 to 55 kg. The animals were not fed for 16 hours before the procedure but allowed water *ad lib*. Anesthesia was induced with ketamine (20 mg/kg intramuscularly) and xylazine (2 mg/kg intramuscularly). After endotracheal intubation, the ventilator was adjusted to achieve a PCO_2_ between 35 and 40 torr. Anesthesia was maintained with enflurane (2%) and vecuronium (2 mg/kg/h). Normal saline solution (2.5 ml/kg/h) and 10% dextrose solution (1 ml/kg/h) were administered intravenously (IV), and cefazolin was administrated IV for antibiotic prophylaxis. Blood loss was replaced with 10% pentastarch solution (50 ml/kg/h). Femoral venous and arterial catheters were placed for fluid administration, blood sample collection, and arterial pressure monitoring, and a double lumen catheter was inserted into the internal jugular vein for BAL attachment. Cystostomy was performed to measure urine output. A hole was drilled through the frontal bone and a probe was placed in the epidural space for intermittent measurement of intracranial pressure (ICP).

Porcine model of ALF was generated by a complete hepatic inflow devascularization procedure. First, the portal vein and inferior vena cava were partially clamped and a side-to-side portocaval shunt was placed before portal vein ligation. Complete ligation and dissection of the hepatoduodenal ligament was followed by dissection of all ligaments around the liver. Running continuous prolene 4–0 sutures were done along the caudate lobe to ensure devascularization of the liver adjacent to the inferior vena cava.

Following surgery, animals were kept under optimal intensive care which included precise monitoring of liver function, urine output, hemodynamic parameters, and ICP until death. Body temperatures were maintained between 38 and 39 °C using a heating blanket.

An animal was considered to have “died” when a mean blood pressure below 30 mm Hg persisted for over 2 hours.

### Experimental design

Animals that underwent the hepatic devascularization procedure were divided into 3 groups: treatment naïve control group (control group; n = 5), the blank treatment group (blank group; n = 5) and the BAL treated group (BAL group; n = 5) groups. BAL group animals were treated by loading the BAL system with 20 billion hepatocytes. In order to assess the impact of extracorporeal circulation and the role of the bioreactor, blank group animals were treated with alginate beads without hepatocytes. Control animals underwent no extracorporeal circulation. BAL and blank treatments were initiated 3 hours after completing hepatic devascularization and were maintained for 12 hours. Animals were randomly allocated to groups.

### Biochemical analysis

Blood samples were obtained hourly following hepatic devascularization. Blood ammonia (NH_3_), glucose (GLU), albumin (ALB), creatinine (CR), calcium (CA), blood urea nitrogen (BUN), aspartate aminotransferase (AST), alanine aminotransferase (ALT), total bilirubin (TBIL), lactate dehydrogenase (LDH) and alkaline phosphatase (ALP) were determined using an automatic biochemical analyzer (Dri-chem 3000, Fujifilm, Japan). Blood electrolytes and pH levels were measured using an arterial blood gas analyzer (Rapidlab 865, Bayer, Germany), and activated clotting times (ACT) were determined using a whole blood coagulation system (Hemochron, ITC, USA).

### Statistical analysis

Survival rates were estimated using the Kaplan-Meier method and the log-rank test was applied to compare survival distribution between three groups. A generalized estimating equation (GEE) was used for comparison of trend over time between three groups and Bonferroni’s correction was used to adjust p-value for multiple comparisons. Statistical analyses were executed using SAS version 9.4 (SAS Institute, Cary, NC).
